# Evaluating the scope and impact of pre-diagnostic manipulative therapy in children and adolescents with osteosarcoma: A retrospective study in Uganda

**DOI:** 10.1371/journal.pone.0329688

**Published:** 2025-08-04

**Authors:** Richard Nyeko, Fadhil Geriga, Racheal Angom, Joyce Balagadde Kambugu, Jaques van Heerden

**Affiliations:** 1 Department of Paediatrics and Child Health, Lira University, Lira, Uganda; 2 Division of Paediatric Oncology, Uganda Cancer Institute, Kampala, Uganda; 3 Department of Paediatric Haemato-Oncology, Antwerp University Hospital, University of Antwerp, Antwerp, Belgium; Shuguang Hospital Affiliated to Shanghai University of Traditional Chinese Medicine, CHINA

## Abstract

**Background:**

Pre-diagnosis manipulative therapies in patients diagnosed with osteosarcoma can compromise patients’ outcomes. Limited literature exists on the pre-diagnosis non-oncological management of osteosarcoma, especially in resource-limited settings. We described and characterized the practice of pre-diagnosis manipulative therapy at the reference cancer treatment center in Uganda as a first step to improving the quality along the osteosarcoma treatment pathway.

**Methods:**

We reviewed the demographic and clinical characteristics, pre-referral management, and outcome of children under 18 years treated for osteosarcoma at the Uganda Cancer Institute between January 2016 and December 2020. Data on pre-diagnosis management were extracted, as well as clinical and disease characteristics and outcome. Descriptive statistics and Kaplan-Meier survival analysis were used.

**Results:**

Nineteen (25.7%) of the 74 children with osteosarcoma in the current study had undergone prior manipulative therapy. The main forms of manipulative therapy were local therapeutic cuttings with the application of local herbs in 6 (31.5%) patients, massaging in 5 (26.3%), attempted incision and drainage in 4 (21.1%), and treatment as a local infection (osteomyelitis or arthritis) in 4 (21.2%). The majority, 15 (78.9%), of the patients who had manipulative therapy were males (X² = 5.73; p = 0.031). Children who underwent manipulative therapy were referred after a median of 45 days (range 11–139) compared to their counterparts, who were referred after a median of 28 days (range 1–147) (p = 0.012). Patients with manipulative therapy had a metastatic rate of 77.8%, while the rate was 60.4% for those who did not have manipulative therapy. The serum lactate dehydrogenase level was higher in patients who had manipulative therapy compared to those who did not have manipulative therapy (X^2^ = 3.98; p = 0.046). The median survival was 1.0 year (95% CI 0.8–1.3) for patients who underwent prior manipulative therapy and 1.8 years (95% CI 1.4–2.2) for those who did not report any form of manipulative therapy (p = 0.961).

**Conclusion:**

Pre-diagnosis manipulative therapies lead to poorer outcomes in patients diagnosed with osteosarcoma, underscoring the need for bridging the quality gap in the osteosarcoma treatment pathway. This should include sensitization of the community and healthcare provider’s, strengthening patient referral pathways, and improved accessibility to cancer treatment centers.

## Background

Osteosarcoma is the most common primary malignant bone tumor of children and adolescents, originating from the primitive mesenchymal cells that produce bones [1]. Yet the incidence of osteosarcoma is only 5–7 cases/million/year [2], with limited from Africa and other low- and middle-income countries (LMICs).

Cancer treatment has significantly evolved over time from surgery as the first rational cancer treatment modality prior to the 1970s, and subsequently radiation therapy, chemotherapy, hematopoietic stem cell transplantation, and others. The period between 1970 and the current saw the development of pharmacological hormonal therapy, targeted therapy, and immunotherapy [3]. Emerging directions in oncology have focused on four key areas, including technical perspectives that emphasize the use of multidisciplinary teams’ management, nutritional perspectives, psychological perspectives, and newer therapies and preventive oncology—with significant advances in stem cell therapy, targeted therapy, ablation therapy, nanoparticle therapy, and natural antioxidants, among others [4,5]. While these modalities will continue to shape cancer therapy for the foreseeable future, multiple approaches are likely to be needed for cancer therapy for the foreseeable future [3].

With the current standard treatment for osteosarcoma, preoperative neoadjuvant chemotherapy, local control achieved through tumor resection with surgical margin, and postoperative adjuvant chemotherapy, 5-year survival rates for localized disease of approximately 60–70% can be achieved [6]. By contrast, long-term survival rates with a surgery-only approach prior to the introduction of chemotherapy were less than 20% [7,8].

However, the outcome of osteosarcoma remains low in LMICs, occasioned by late presentation and advanced disease at diagnosis, misdiagnosis and treatment, among others. The combination of the sites of tumor location, commonly the distal femur [9] and proximal tibia, and association with swelling and pain may often lead to misdiagnosis and initial incorrect management of osteosarcoma with detrimental effect on chances of limb-salvage surgery and survival. In Africa, traditional health care-seeking practices and low awareness of childhood cancers lead to the use of alternative and complementary treatments prior to a cancer diagnosis [10–14]. In fact, traditional and complementary medicine (TCM) has gained wide use in clinical cancer treatment in many countries [15,16], the benefits of which are believed to include direct therapeutic effect, reduction of side effects, and prevention of cancer, among others [17]. Such therapies, including massage manipulations on the tumor site, have been suggested to result in micro-metastasis due to hyper-vascularization [18–20]. This has the potential to compromise especially limb-salvage surgery outcomes and lead to a poorer survival rate [20]. Likewise, as Heng-Rui Liu asserts, the potential side effects and interactions of TCM with other therapies, particularly cancer treatments, warrant careful consideration, despite being generally considered safe, especially when used in low doses and under proper guidance [15].

In Uganda, most children and adolescents with osteosarcoma present late—upto one-half with metastatic disease [21]. A potential underlying factor is the non-referral from previous medical appointments with patients undergoing some form of manipulative therapy prior to diagnosis. While this defies the standard of care for osteosarcoma with a possible negative effect on treatment and outcome, these practices and their impact are not documented [18,19]. This study, therefore, aimed, to describe and characterize the practice of pre-diagnosis manipulative therapy as a first step to galvanize action to improve the osteosarcoma treatment pathway.

## Methods

### Study design and setting

This was a retrospective study that reviewed records to assess children and adolescents under 18 years with osteosarcoma treated at the Uganda Cancer Institute (UCI) from January 2016 (the time point from which records for children were accessible) to December 2020. The UCI is a 200-bed capacity facility, 43 of which are dedicated to children and adolescent inpatients, and it is Uganda’s only national reference cancer treatment center. Nearly 80% of children with cancer in the country are treated at the UCI, where about 400–500 new childhood cancer cases are seen annually, making this a representative site in the country for conducting the study.

### Study population

**Fig 1:**
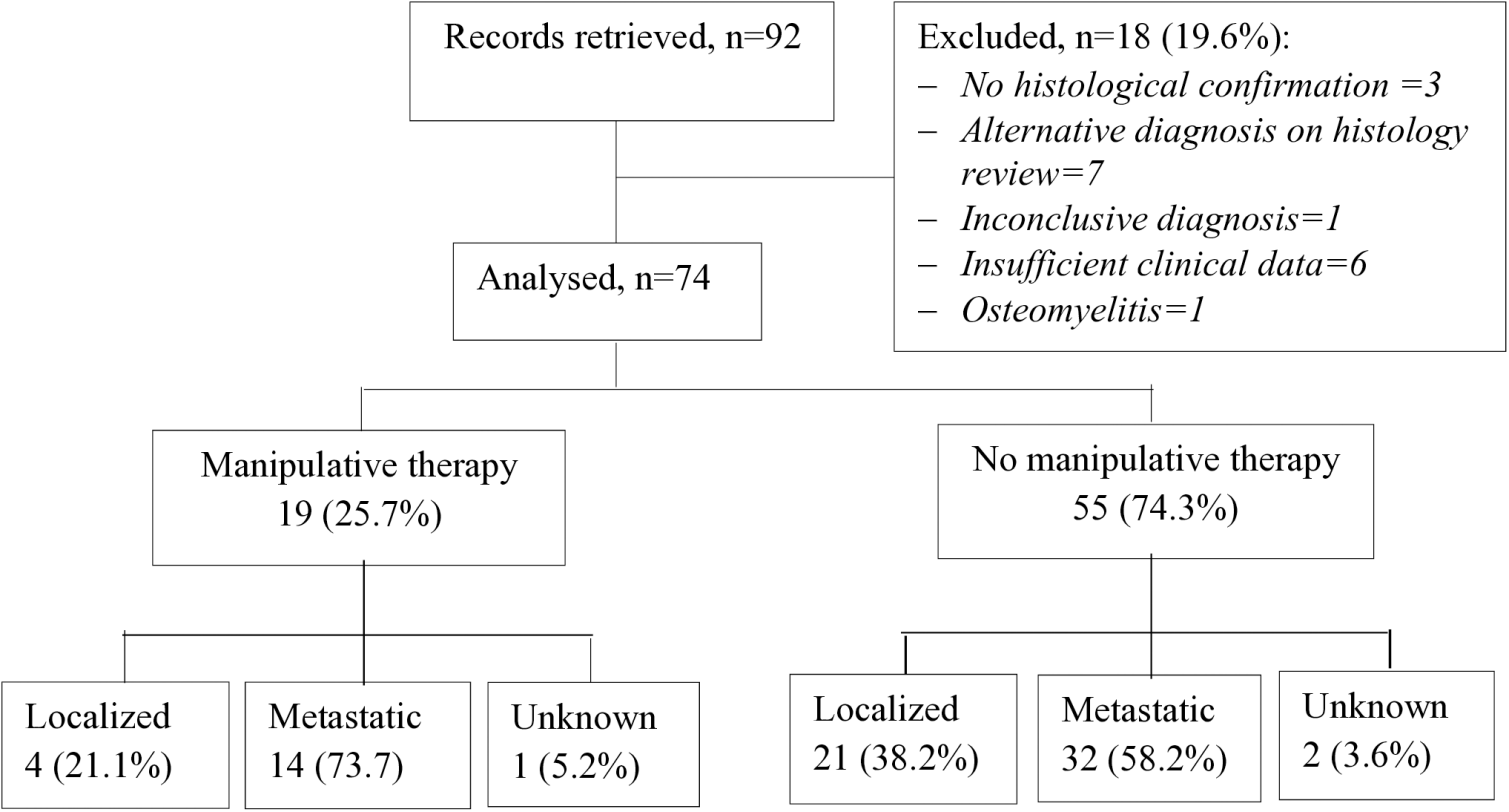
Study flow diagram.

The study included children and adolescents under 18 years of age treated for osteosarcoma at the study site within the study period. Patients with an uncertain or inconclusive diagnosis, incomplete medical records lacking clinical details, or an alternative diagnosis on histology review were excluded (Fig 1).

### Clinical and outcome definitions

Manipulative therapy was defined as therapy that was administered to a patient due to the misdiagnosis of the osteosarcoma that could be potentially harmful to the tumor and lead to poorer management outcomes of the tumor diagnosis. These include incision and drainage, cuttings over the tumor lesion from traditional healers, and massage maneuvers, among others.

Overall survival (OS) was defined as the time duration from the date of diagnosis to death from any cause or to the date the patient was last known to be alive.

### Study procedure and data extraction

We accessed the records and collected data for this study from June 15, 2022, to September 30, 2022. All accessible patient records within the study period were consecutively retrieved and reviewed. Individual patients’ data were extracted from the time of cancer diagnosis to the date the patient was last seen in the clinic or died. Demographic information (age and sex/gender), duration of symptoms, pre-referral interventions, disease characteristics, patient management, and clinical outcome were collected.

### Osteosarcoma diagnosis and treatment

The diagnosis of osteosarcoma was made based on clinical presentations and radiological findings and confirmed by histological examination of tissue biopsy based on the morphologic criteria defined by the World Health Organization (WHO) classification [22]. The primary site and local extent of the tumor were assessed by a computed tomography (CT) scan or plain X-ray in some cases (where a CT scan was not accessible). The initial staging workup included a CT scan of the chest and a skeletal survey. Radionuclide technetium-99m (Tc-99m) scan during staging was not available.

All children with osteosarcoma included in the study were treated according to the local standard protocol, which is based on a two-drug combination chemotherapy regimen with Cisplatin and Adriamycin (AP) that does not involve high-dose methotrexate. The chemotherapeutic regimen entailed three courses of neoadjuvant induction chemotherapy and two courses of adjuvant maintenance chemotherapy administered every 21 days, regardless of histologic response to chemotherapy (*see additional file 1*). Interval radiological evaluations were performed prior to surgery, and individuals with resectable tumors were offered local control, often radical surgery (amputation or disarticulation). Physical examination, radiographic investigations, and biopsy, where feasible and appropriate, are used to confirm recurrences (local or systemic).

### Data management and statistical analysis

Data were analyzed using the Statistical Package for Social Sciences (SPSS) software package (SPSS for Windows, Version 27, Chicago, SPSS Inc.). Descriptive statistics for categorical variables were presented as frequencies and percentages, while continuous variables were summarized as means with standard deviation if normally distributed or medians with interquartile range if non-normally distributed. Median survival, with the associated 95% confidence intervals (CI), was estimated using the Kaplan-Meier method and compared using the log-rank test [23]. The Kaplan-Meier plot is a graphical method used to estimate the probability of survival over time. It is especially useful for visualizing survival differences between groups. The log-rank test is a statistical test used to compare the survival curves of two or more groups. The test assesses whether there are significant differences in survival times between the groups, taking into account censoring. A p-value from the log-rank test below a predetermined significance level, usually 0.05, indicates a statistically significant difference in survival between the groups [24]. Statistical significance was defined as a p-value <0.05.

### Ethics approval and consent to participate

All methods were carried out in accordance with relevant guidelines and regulations and the study was conducted in accordance with the Declaration of Helsinki. The study was approved by the Uganda Cancer Institute Research and Ethics Committee (UCI-2022–44). Written informed consent and assent were accordingly waived by the research and ethics committee. Despite the presence of identifiers like participants’ names in the patients’ records, the study used codes in place of these identifiers to maintain confidentiality during data collection and throughout the study.

## Results

### Description of study participants

Of the 74 children and adolescents with osteosarcoma identified within the study period, 19 had undergone manipulative therapy prior to referral to the cancer treatment center (Fig 1).

**Fig 2:**
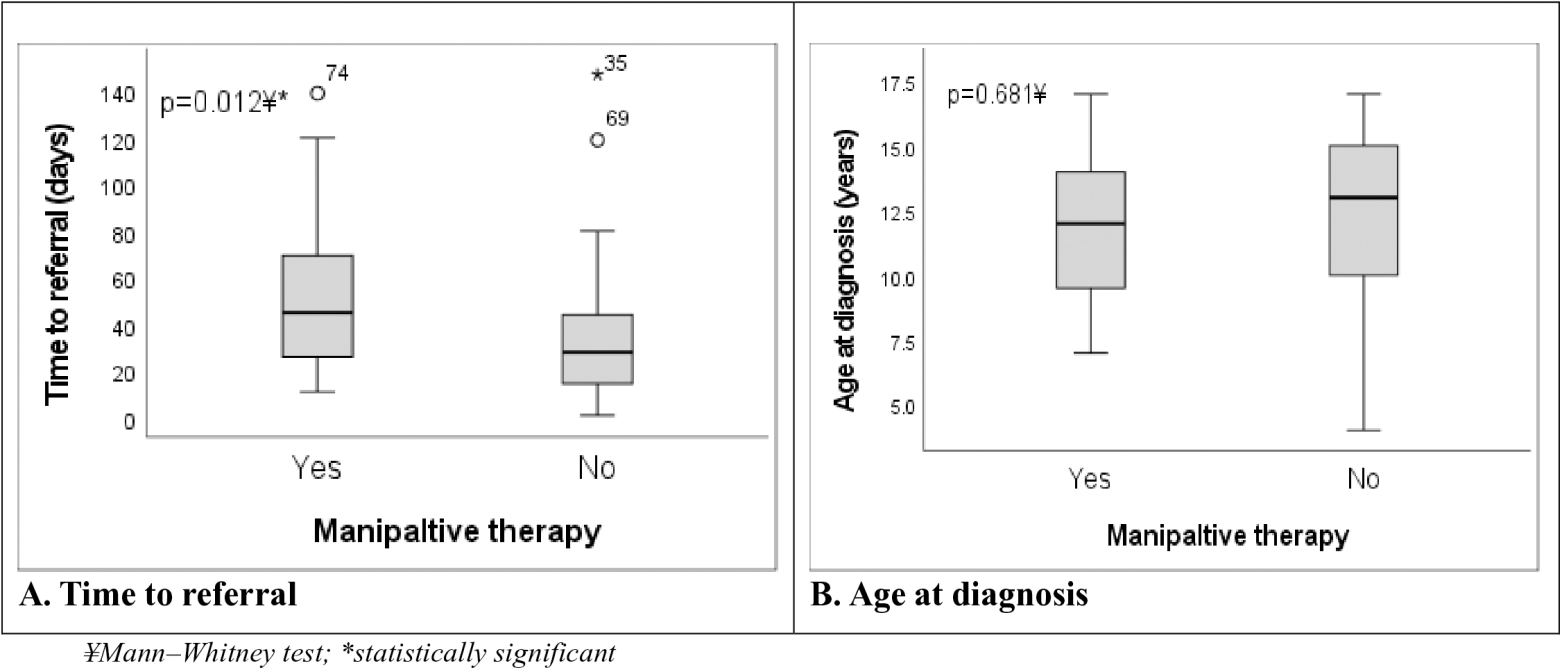
Box plots of time to referral and age at diagnosis by manipulative therapy status in children with osteosarcoma.

Table 1 presents the demographic and clinical characteristics of the group who had undergone manipulative therapy and the group who had not. There was a statistically significant association between manipulative therapy and sex as well as timeliness of referral. Patients who underwent manipulative therapy were significantly more likely to be males, 15/19 (78.9%), than females, 4/19 (21.1%) (χ^2 ^= 5.73; p = 0.031). Referral was delayed (≥28 days) in 13 (68.4%) and early (<28 days) in only 6 (31.6%) of the patients who underwent manipulative therapy (χ^2 ^= 4.02; p = 0.043). In fact, children who underwent manipulative therapy were referred after a median of 45 days (range 11–139) compared to their counterparts who were referred after a median of 28 days (range 1–147) (Fig 2A). There was no statistically significant difference in the other patient characteristics (age, B symptoms [fever, drenching night sweats, and weight loss], pain symptoms, and history of trauma) with regards to manipulative therapy (Table 1). However, patients who had manipulative therapy were relatively younger (median age 12.0 years; IQR 9–15) than their counterparts (median age 13.0 years; IQR 10–15) (p = 0.681) (Fig 2B).

**Table 1:** Demographic and clinical characteristics.

Variable	Manipulative therapy		χ^2^	p value
	Yes (n = 19)	No (n = 55)		
**Age at diagnosis (years)**				
≤12	10 (52.6)	26 (47.3)	0.16	0.687
13-17	9 (47.4)	29 (52.7)		
**Sex**				
Male	15 (78.9)	26 (47.3)	5.73	0.031*
Female	4 (21.1)	29 (52.7)		
**Time to referral to cancer treatment center**				
<28 days	6 (31.6)	26 (59.1)	4.02	0.043*
≥28 days	13 (68.4)	18 (40.9)		
**B symptoms**				
Yes	13 (68.4)	26 (47.3)	2.53	0.108
No	6 (31.6)	29 (52.7)		
**Pain**				
Yes	15 (78.9)	44 (80.0)	0.01	0.922
No	4 (21.1)	11 (20.0)		
**History of trauma**				
Yes	7 (36.8)	15 (27.3)	0.62	0.437
No	12 (63.2)	40 (72.7)		

χ^*2*^ *Chi-square (1 degree of freedom); B symptoms = fever, drenching night sweats and unexplained weight loss.*

### Prevalence and forms of manipulative therapy among children with osteosarcoma

**Fig 3:**
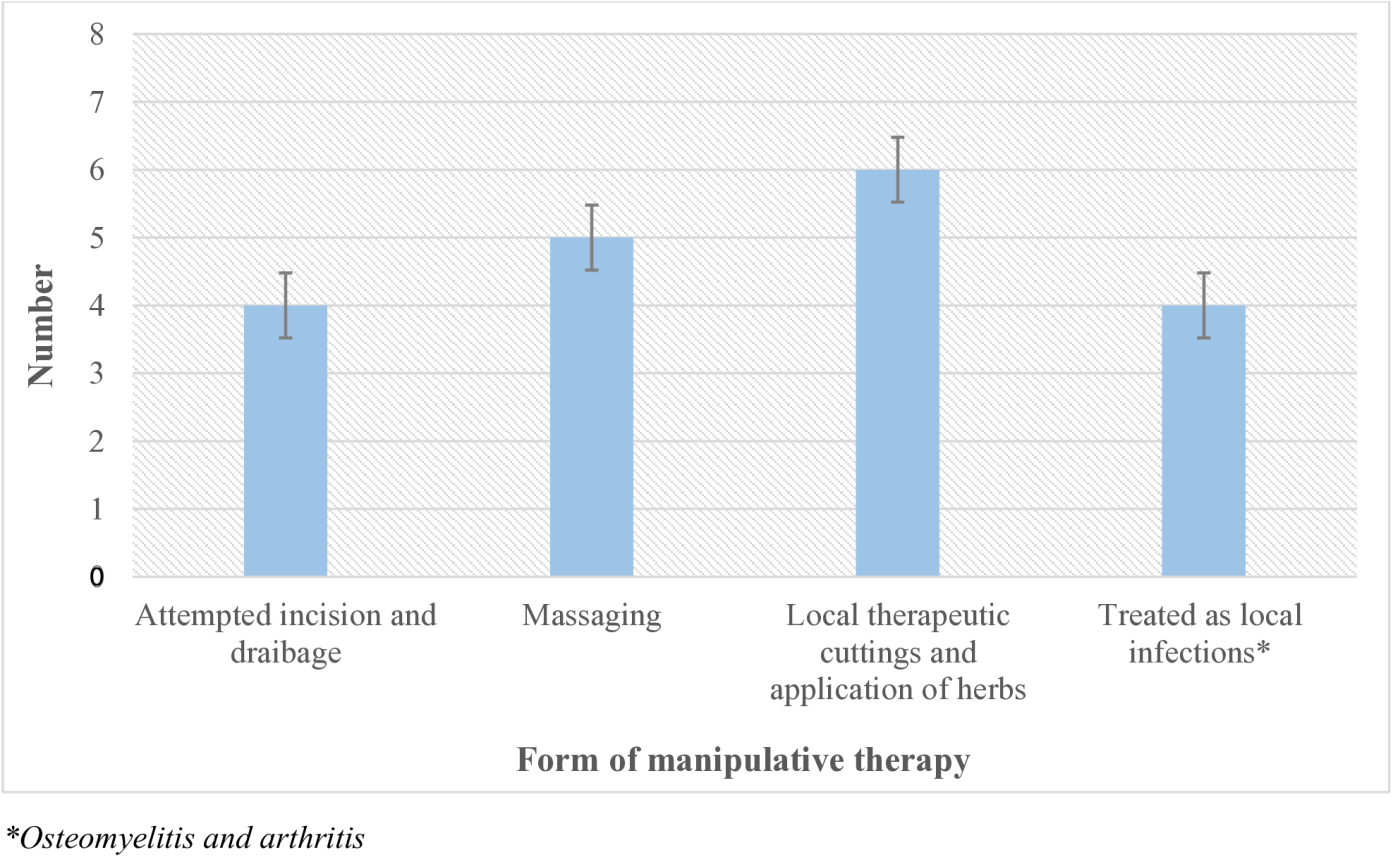
Forms of manipulative therapy among children with osteosarcoma.

Nineteen of the 74 children and adolescents with osteosarcoma in the current study had undergone manipulative therapy prior to referral to the cancer treatment center, giving a prevalence of 25.7%. The majority, 6 (31.5%), had local therapeutic cuttings with the application of local herbs, followed by massaging, 5 (26.3%); attempted incision and drainage, 4 (21.1%); and treatment as local infections (osteomyelitis), 4 (21.2%) (Fig 3).

### Level of facility where biopsy was performed and disease metastasis

**Fig 4:**
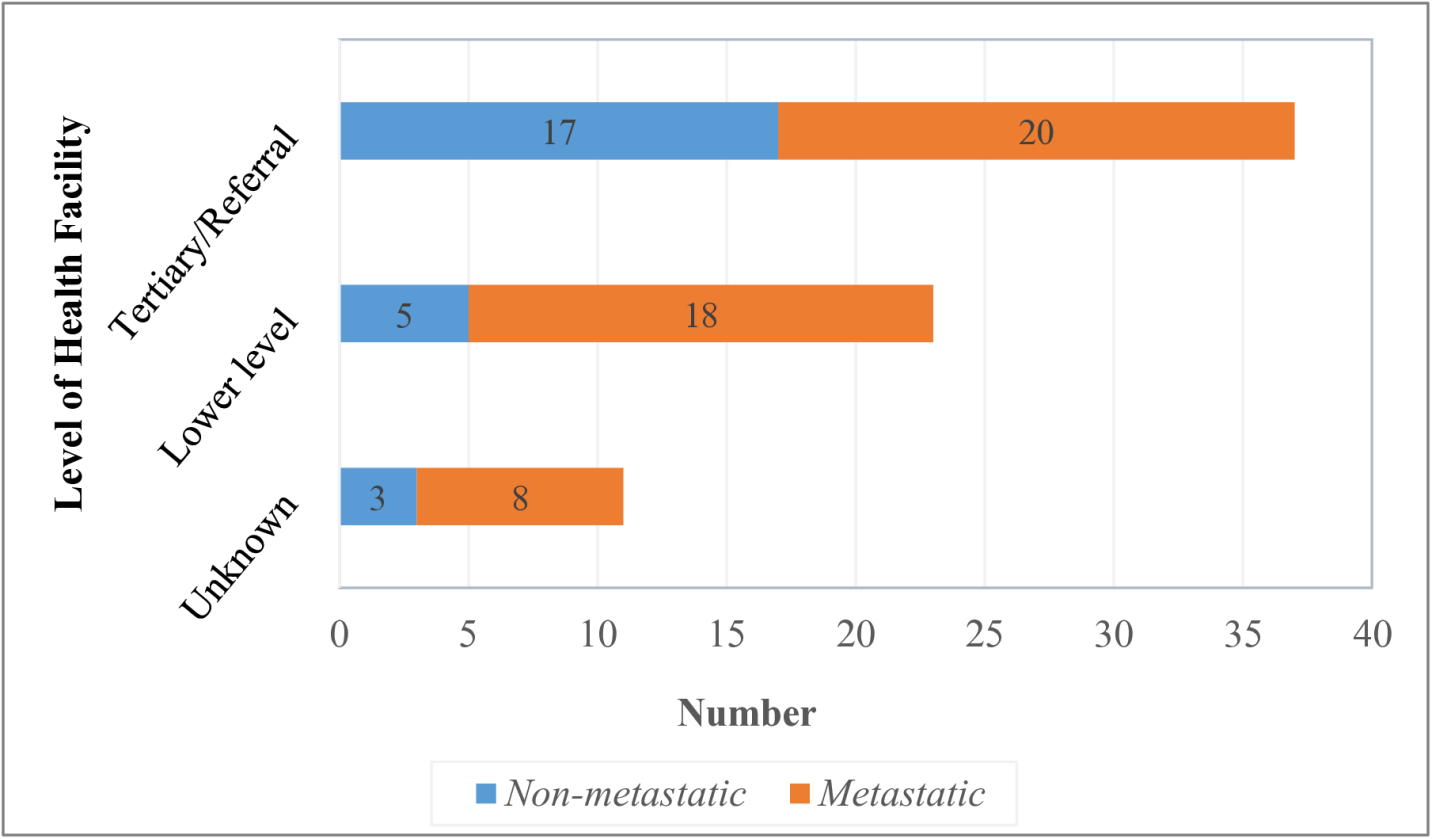
Level of facility where biopsy was performed and disease metastasis.

Majority, 40 (54.0%) of the biopsies were performed at tertiary referral facilities, and 23 (31.1%) at lower-level health facilities (district hospitals, medical centers, and private facilities). The rate of metastatic disease was 52.5% (21/40) among the patients who had a biopsy from tertiary health facilities and 78.3% (18/23) among patients who had a biopsy from lower-level health facilities. The facility of biopsy was undocumented in 11 (14.9%) of the patients, 72.7% (8/11) of whom had metastatic disease (Fig 4).

### Profile and outcomes of patients who had manipulative therapy

Patients in whom manipulative therapy was performed largely had osteosarcoma primaries in the limbs, mainly the lower limbs. Information on the size of the primary tumor was available for 16 patients, with the median primary tumor size of 15.0 cm (range 5–25) (Table 2).

**Table 2:** Demographic and clinical profile and outcomes of patients who had manipulative therapy.

SN	Age	Sex	Primary site	Size (cm)	Trauma	Metastasis	Surgery	Timing of surgery	Status
1	15	Male	Proximal tibia/fibula	18	No	–	No	–	Died
2	12	Male	Distal femur	12	Yes	Yes	Yes	Upfront	Died
3	15	Female	Proximal tibia	5	No	No	Yes	Neoadjuvant	Alive
4	12	Female	Distal femur	12	No	No	Yes	Upfront	Alive
5	9	Female	Hip	–	No	Yes	No	–	Alive
6	13	Male	Distal femur	15	No	Yes	No	–	Died
7	7	Male	Distal femur	–	No	Yes	Yes	Upfront	Alive
8	13	Male	Distal femur	–	Yes	No	Yes	Upfront	Alive
9	8	Male	Distal femur	22	No	No	No	–	Died
10	7	Male	Distal femur	20	No	Yes	No	–	Died
11	7	Male	Distal femur	10	Yes	Yes	No	–	Died
12	12	Female	Proximal femur	6	No	Yes	No	–	Died
13	10	Male	Distal femur	–	No	Yes	Yes	Neoadjuvant	Died
14	13	Male	Proximal tibia	–	Yes	Yes	Yes	Upfront	Died
15	11	Male	Distal femur	25	Yes	Yes	Yes	Upfront	Died
16	15	Male	Proximal tibia	–	Yes	Yes	Yes	Upfront	Died
17	13	Male	Proximal tibia	11	No	Yes	Yes	Upfront	Died
18	17	Male	Proximal humerus	20	Yes	Yes	No	–	Died
19	15	Male	Proximal tibia	20	No	Yes	Yes	Upfront	Died

*SN. Serial Number.*

### Disease and laboratory characteristics

**Fig 5:**
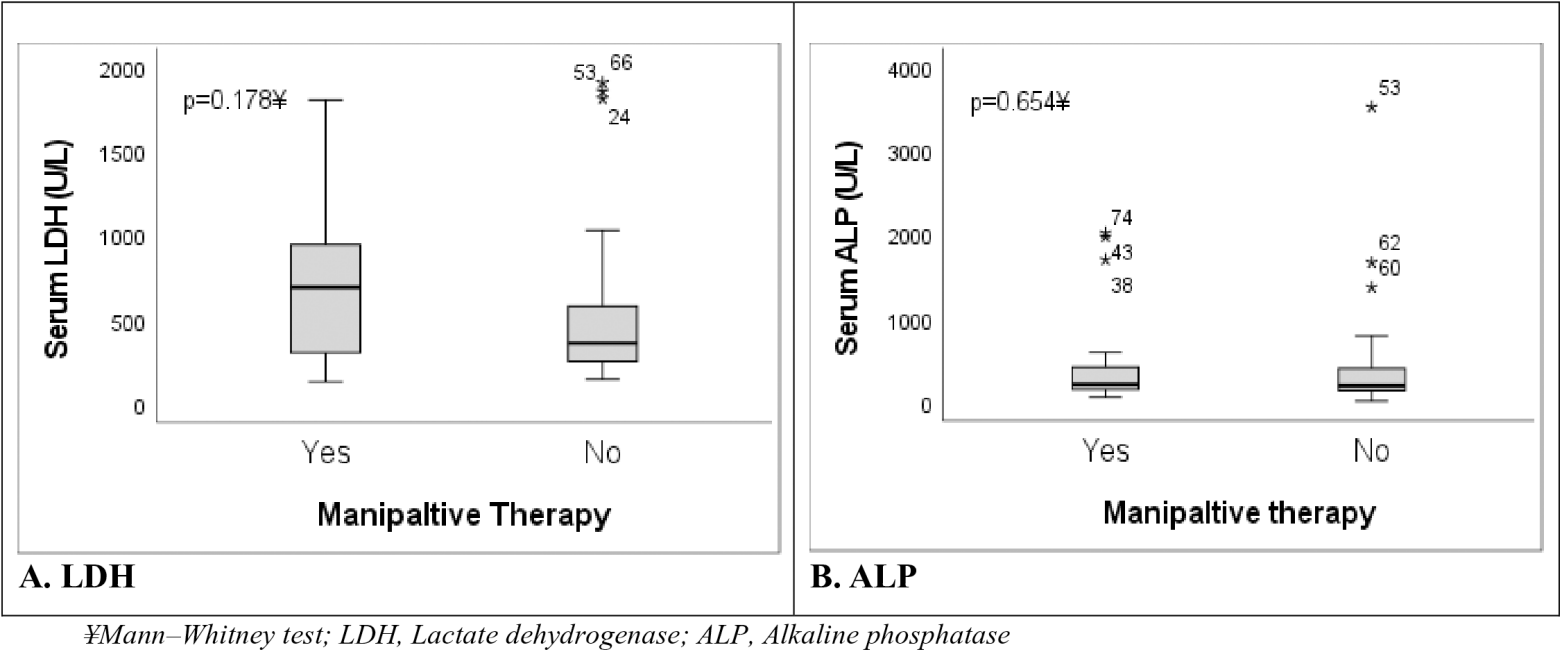
Box plots of serum LDH and ALP levels by manipulative therapy status in children with osteosarcoma.

There was a statistically significant association between manipulative therapy and serum LDH level. Close to two-thirds, 11 (64.7%) of the patients who underwent manipulative therapy, had high serum lactate dehydrogenase (LDH) levels (>500 U/L), and just over a third, 6 (35.3%), had serum LDH < 500 (X^2 ^= 3.98; p = 0.046) (Table 3). Likewise, the median serum LDH in children who had manipulative therapy was higher [690 U/L (range 130–1802)] than in those who did not undergo manipulative therapy [359 U/L (range 145–1906)] (Fig 5A) (p = 0.178). Over three-quarters, 14 (77.8%), of the patients who had manipulative therapy presented with metastatic disease, and only 4 (22.2%) had localized disease at diagnosis (X^2 ^= 1.78; p = 0.256). Patients who underwent manipulative therapy commonly had upfront surgery (81.8%; n = 9) rather than surgery after neoadjuvant chemotherapy (18.2%, n = 2), although this difference was not statistically significant (X^2 ^= 1.39; p = 0.291). (Table 3). Serum alkaline phosphatase (ALP) was > 150 U/L in 13 (72.2%) and ≤150 U/L in 5 (27.8%) of the patients who had manipulative therapy (X^2 ^= 0.29; p = 0.588). Relatedly, there was a tendency for higher serum ALP levels (median 209 U/L; IQR 142–461) in patients who had manipulative therapy compared to those who did not have manipulative therapy (median 194 U/L; IQR 135–415) (p = 0.654) (Fig 5B).

**Table 3:** Disease and laboratory characteristics.

Variable	Manipulative therapy		χ^2^	p value
	Yes	No		
**Location**				
Tibia	5 (26.3)	11 (20.0)	0.33	0.570
Other sites	14 (73.7)	44 (80.0)		
**Metastasis**				
Yes	14 (77.8)	32 (60.4)	1.78	0.256
No	4 (22.2)	21 (39.6)		
**Timing of surgery**				
Upfront	9 (81.8)	20 (62.5)	1.39	0.291
After neoadjuvant therapy	2 (18.2)	12 (37.5)		
**LDH (U/L)**				
≤500	6 (35.3)	25 (64.1)	3.98	0.046*
>500	11 (64.7)	14 (35.9)		
**ALP (U/L)**				
≤150	5 (27.8)	16 (34.8)	0.29	0.588
>150	13 (72.2)	30 (65.2)		
**Serum albumin (mg/dL)**				
Normal	12 (63.2)	34 (63.0)	0.00	0.988
Low	7 (36.8)	12 (37.0)		

χ^*2*^ *Chi-square (1 degree of freedom);* ^*§*^*three had incomplete metastatic assessment and were excluded;* ^*¶*^*Only those who underwent surgical resection.*

### Manipulative therapy and survival outcomes

**Fig 6:**
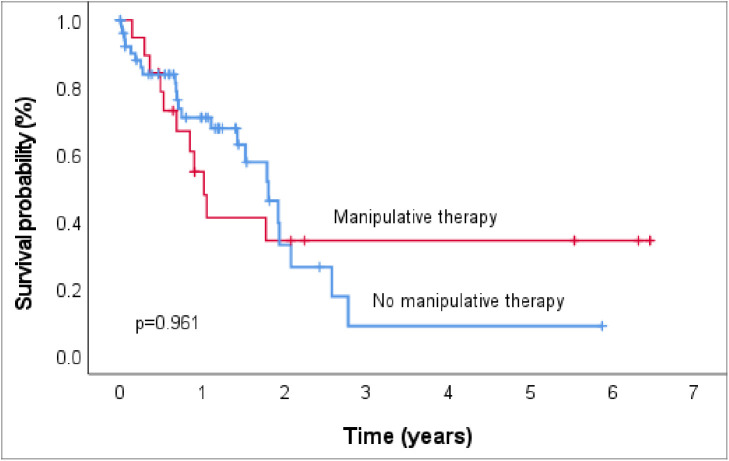
Overall survival of children with osteosarcoma with regard to manipulative therapy.

There was no statistically significant difference in overall survival (OS) between the two groups of patients with respect to manipulative therapy. The median survival for patients who underwent prior manipulative therapy was 1.0 year (95% CI 0.8–1.3), and it was 1.8 years (95% CI 1.4–2.2) for those who did not undergo any form of manipulative therapy (p = 0.961) (Fig 6). We did not demonstrate interaction between manipulative therapy and time to initiation of therapy (p = 0.343) in view of the fact that manipulative therapy was associated with delayed referrals, and yet time to initiation of therapy also adversely affects prognosis.

## Discussion

Pre-diagnosis manipulative therapy in children with osteosarcoma is an unexplored problem, especially in resource-limited settings, where the healthcare system is challenging. However, in a recent review examining the challenges of osteosarcoma care in Africa, unorthodox medicine practitioners were noted to represent a significant barrier contributing to poor outcomes in the region [25] Our study demonstrated that pre-diagnostic manipulative therapy is prevalent among our child and adolescent populations with osteosarcoma and also indicated the characteristics associated with this practice, although the true impact on survival is unclear.

In this study, a third of the children and adolescents with osteosarcoma had undergone manipulative therapy before they were referred to the cancer treatment center, which may characterize traditional health-seeking behaviors of Ugandans. The rate in our study is much lower than the 52% and 69% reported among patients with osteosarcoma in Asian countries [18,26], a difference that could be attributed to the nature of manipulative therapies and the age groups considered. One of the Asian studies [26] only considered massage therapy—a practice that is possibly more common among adults and in eastern Asia. The current study’s findings are clinically significant and may indicate high rates of misdiagnosis and mismanagement, adversely impacting osteosarcoma treatment and outcomes in resource-limited settings. Yao and Lin highlighted that the non-specific presentation of osteosarcoma in its early stages, absence of typical features on X-ray, and insufficient clinical knowledge among physicians, significantly contributes to misdiagnosis and inappropriate treatment [27]— underscoring the necessity for training and skill enhancement among primary healthcare providers.

The forms of manipulative therapy before referral were varied but largely involved locally invasive procedures, including local therapeutic cuttings over the tumor swelling, incision and drainage, and massage therapy. In a Chinese study, the major forms of mistreatment included massage therapy (29.8%), surgical resection for benign tumors or local injury (23.4%), and use of traditional Chinese medicine (29.8%), among others [27]. In addition, malignant lesions are commonly diagnosed as inflammatory disease [27]. The misdiagnosis of a malignant lesion as an abscess and hence attempting incision and drainage, just as the practice of local cuts over the tumor lesions and application of local herbs, as found in the current study, can have significant negative effects on the management and prognosis of these patients [19]. It is important, therefore, that clinicians, especially at the primary healthcare level, be empowered with the necessary knowledge to have a heightened level of suspicion and pay more attention to the clinical symptom of osteosarcoma [19,27].

Manipulation of tumors is not only limited to traditional or holistic health-seeking behavior. Erroneous medical practices, such as tumor biopsies performed by unqualified persons, equally affect management outcomes. A poorly placed biopsy track may result in the spread of the tumor cells, as well as damage to vital structures [28]. This may necessitate a more extensive resection, reducing reconstructive options and compromising possible limb salvage, leading to an amputation instead [29,30]. Likewise, contaminated limb compartments increase the risk of residual tumor after resection and an increased risk for local recurrence [30]. In the current study, patients who had a biopsy performed at lower-level facilities—about a third of the study participants—had a higher rate of metastatic disease compared to those who had a biopsy at tertiary care facilities. While we could not determine the temporality in this case and it may not be causal, this finding is contrary to the ideal that biopsy in osteosarcoma should be performed at the center where the definitive treatment is to be performed under the guidance of a well-trained oncologist, following the basic principles [29,31,32]. The possibility that some of the biopsies, especially those from lower-level health facilities, may not be based on oncological principles should therefore be explored and addressed if any.

Prior manipulative therapy in the current study was associated with a longer time to referral to the cancer treatment center for appropriate diagnosis and treatment. This compounds a major challenge in cancer management in LMICs: late presentation and referral leading to advanced disease stage and poor outcome [33]. Misdiagnosis and mistreatment not only delay the initiation of the best treatment at the best opportune time but also stimulate the growth of tumor cells directly as well as disease metastasis, resulting in more advanced stages of disease at presentation [27,34]. While poor health systems and referral pathways, as well as challenges in accessibility to the few cancer treatment centers, could be some of the contributing factors, lack of knowledge about the symptoms and signs and the diagnostic and therapeutic principles of osteosarcoma among the traditional healers and primary healthcare providers alike could have accounted to delayed referral in the current study context [21,27]. Underlying this is the lack of a specific policy and focused training initiatives on childhood cancer in general. This underscores the need for a concerted initiative towards raising community awareness and building the capacity of first-level health providers and traditional healers [19], including community outreach and a simple referral checklist for primary healthcare providers.

Available pieces of evidence suggest that pre-diagnostic manipulative procedures pose a risk of damaging the tumor and forming micro-metastasis due to hyper-vascularization, as demonstrated in an in vivo laboratory-based study by Wang et al. [19] with the potential to compromise the chances for limb salvage surgery [14] and the overall patient outcome [19,26]. Our result is in concurrence with the above, showing an increased rate of metastasis in association with manipulative therapy than in patients who did not report manipulative therapy. This finding replicates findings reported by Karda et al. in Indonesia, where message manipulation increased the risk of metastasis in patients with osteosarcoma [26]. The negative effect of manipulative therapy on the risks of disease metastasis was also evident in a study by Wu et al., where prior manipulative therapy resulted in significantly higher rates of both primary (32% vs. 3%) and overall (51.4% vs. 18.8%) pulmonary metastases [18]. This is an important observation given that metastasis is a poor prognostic factor associated with inferior survival rates in patients with osteosarcoma [21,35,36].

Lactate dehydrogenase (LDH) is an important surrogate for tumor burden and disease extent. In this study, the serum LDH level was significantly higher in patients who had prior manipulative therapy compared to the level in those who did not report manipulative therapy. Likewise, there was a trend towards higher serum alkaline phosphatase (ALP) levels in association with prior manipulative therapy. This finding is in keeping with that reported among children and adults with osteosarcoma in Indonesia, where three-quarters of the patients who had massage therapy had raised LDH levels [26]. Furthermore, our study correlates the relation between massage therapy and increased LDH and ALP levels in patients with osteosarcoma in China [18]. Raised serum LDH has been shown to portend an inferior prognostic outcome and is significantly associated with poor survival outcomes [37,38] and high relapse [39].

The median overall survival (OS) for patients who underwent pre-referral manipulative therapy was lower compared to those who reported no prior manipulative therapy. This finding correlates with an Asian study where the five-year OS rate was significantly inferior, with a higher local recurrence rate in patients who had undergone manipulative therapy (58% 5-year OS; 29% recurrence rate) compared to the groups without manipulative therapy (92% 5-year OS; 6% recurrence rate) [18]. Similar findings have been reported among patients who underwent unplanned treatment—for whom the 5-year survival rate was lower (60%) than for patients who received planned treatment (75%) [40]. This finding could likely be related to the attendant delay in referral with advanced disease stage and the risks of metastasis associated with locally invasive manipulative and unplanned therapies [19,26,27]. Although manipulative therapy was associated with delayed referral, and because time to initiation of therapy adversely affects prognosis, we did not find an interaction between these two variables, which reaffirms the effect of manipulative therapy on the observed poorer survival outcome. Importantly as well, there was a generally poor survival outcome even for patients who did not have manipulative therapy, which could be attributed to more advanced disease at presentation, lack of local control, and the high rate of treatment abandonment (over a third of the patients). Likewise, manipulative therapy could have also contributed to the observed treatment abandonment—by compromising treatment outcomes, in addition to inflicting bad experiences, making patients or their caregivers more reluctant to return. Conversely, patients may also abandon conventional cancer therapies for the unconventional manipulative maneuvers [41].

The limitation of this study is the possibility of reporting bias of any prior manipulation therapy, especially given that some of these were undocumented, some of which, like local cuttings and use of herbs, are believed to carry negative connotations in the conventional healthcare system. Likewise, the study was conducted in only one oncology unit in Uganda, and though the findings may not necessarily be generalizable to the whole region or other contexts, it underscores the challenges that are prevalent in similar-resource settings. This study was primarily descriptive in nature with a small sample size and was limited in exploring the correlation between manipulative therapy and survival outcomes.

### Conclusion and recommendations

The prevalence of manipulative therapy in the current study setting deserves attention and underscores an overlooked challenge that affects osteosarcoma treatment outcomes in this and other resource-limited settings. Bridging this quality gap in the osteosarcoma treatment pathway should become an area of focus, including sensitization of the community and healthcare providers alike, strengthening patient referral pathways for timely referrals, and improving accessibility to cancer treatment centers and timely oncological interventions. A comprehensive prospective study with a larger sample size is recommended to explore the correlation between manipulative therapy and survival outcomes.

## Supporting information

S1 Fig Additional file 1. (TIF)

S1 File STROBE-checklist-v4-combined-PlosMedicine (PDF)

S2 File Anonymized dataset. (XLSX)

## Abbreviations

HIC: High-income country
IQR: Interquartile range
LIC: Low-income countries
LMIC: Low- and middle-income country
OS: Overall survival
UCI: Uganda Cancer Institute
WHO: World Health Organization
