# Supplementary figures and images for: Evaluating the scope and impact of pre-diagnostic manipulative therapy in children and adolescents with osteosarcoma: A retrospective study in Uganda
Source: PLoS One. 2025 Aug 4;20(8):e0329688. doi: 10.1371/journal.pone.0329688 (PMC12321128; doi:10.1371/journal.pone.0329688)

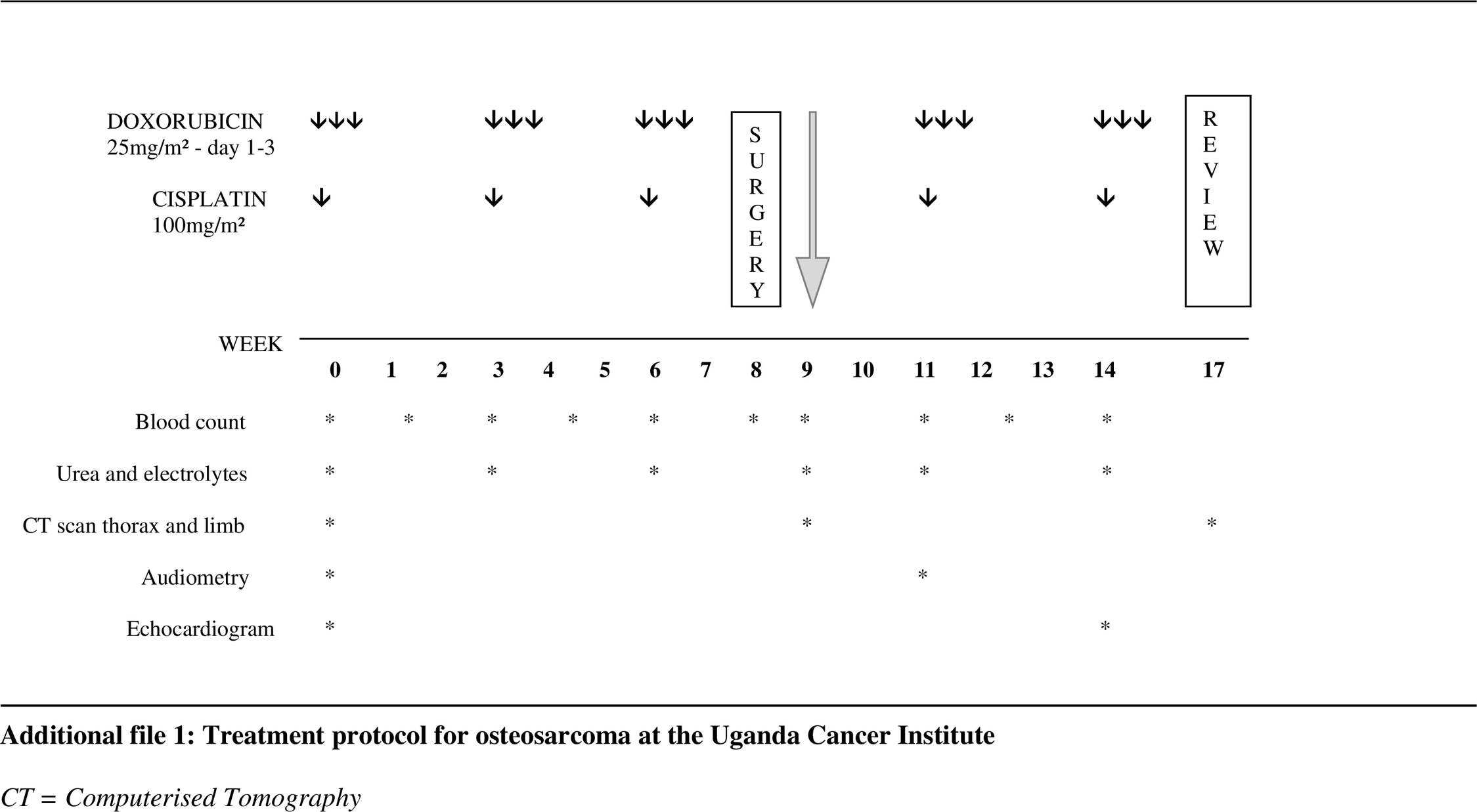

Supplement: S1 Fig — (TIF) [file pone.0329688.s001.tif]
