# Supplementary material for: Evaluating the scope and impact of pre-diagnostic manipulative therapy in children and adolescents with osteosarcoma: A retrospective study in Uganda
Source: PLoS One. 2025 Aug 4;20(8):e0329688. doi: 10.1371/journal.pone.0329688 (PMC12321128; doi:10.1371/journal.pone.0329688)
Supplement: S1 File — (PDF) [file pone.0329688.s002.pdf]

STROBE Statement—checklist of items that should be included in reports of observational studies

	Item No.	Recommendation	Page No.	Relevant text from manuscript
**Title and abstract**	1	(*a*) Indicate the study's design with a commonly used term in the title or the abstract	1	We used a retrospective study design to collect quantitative data
		(*b*) Provide in the abstract an informative and balanced summary of what was done and what was found	2	The abstract of the report provides a summary and an explanation of what was done and the key findings of the study.
**Introduction**				
Background/rationale	2	Explain the scientific background and rationale for the investigation being reported	3-4	The scientific background and rationale of the study is explained in the background section of the report.
Objectives	3	State specific objectives, including any prespecified hypotheses	4	The objective of this study was to describe and characterize the practice of pre-diagnosis manipulative therapy as a first step to galvanize action to improve the osteosarcoma treatment pathway.
**Methods**				
Study design	4	Present key elements of study design early in the paper	4	This was a cross-sectional study design which retrospectively collected quantitative data
Setting	5	Describe the setting, locations, and relevant dates, including periods of recruitment, exposure, follow-up, and data collection	4	This study was conducted at the Uganda Cancer Institute (UCI) from January 2016 to December

				2020. The UCI is a 200-bed capacity facility -43 of which are dedicated to children and adolescent inpatients, and it is Uganda's only national reference cancer treatment center. Nearly 80% of children with cancer in the country are treated at the UCI, where about 400–500 new childhood cancer cases are seen annually, making this a representative site in the country for conducting the study.
Participants	6	(*a*) *Cohort study*—Give the eligibility criteria, and the sources and methods of selection of participants. Describe methods of follow-up<br>*Case-control study*—Give the eligibility criteria, and the sources and methods of case ascertainment and control selection. Give the rationale for the choice of cases and controls	N/A	
		Cross-sectional study—Give the eligibility criteria, and the sources and methods of selection of participants	4 & 5	The study included children and adolescents under 18 years of age treated for osteosarcoma at the study site within the study period. Patients with an uncertain or inconclusive diagnosis, incomplete medical records lacking clinical details, or an alternative diagnosis on histology review were excluded. All accessible patient records within the study period were

				consecutively retrieved and reviewed.
		(*b*)*Cohort study*—For matched studies, give matching criteria and number of exposed and unexposed<br>*Case-control study*—For matched studies, give matching criteria and the number of controls per case	N/A	
Variables	7	Clearly define all outcomes, exposures, predictors, potential confounders, and effect modifiers. Give diagnostic criteria, if applicable	5	The outcome variables were the proportion of children who underwent pre-diagnostic manipulative therapy, and overall survival.<br>The predictor variables were the sociodemographic and disease characteristics.
Data sources/measurement	8*	For each variable of interest, give sources of data and details of methods of assessment (measurement). Describe comparability of assessment methods if there is more than one group	*N/A*	
Bias	9	Describe any efforts to address potential sources of bias	N/A	
Study size	10	Explain how the study size was arrived at	5	The study sample size was based on the number of accessible patients' records within the study period.

Continued on next page

Quantitative variables	11	Explain how quantitative variables were handled in the analyses. If applicable, describe which groupings were chosen and why	6	Descriptive statistics for categorical variables were presented as frequencies and percentages, while continuous variables were summarized as means with standard deviation if normally distributed or medians with interquartile range if non-normally distributed. Median survival, with the associated 95% confidence intervals (CI), was estimated using the Kaplan-Meier method and compared using the log-rank test. Statistical significance was defined as a p-value <0.05.
Statistical methods	12	(*a*) Describe all statistical methods, including those used to control for confounding	6	The report describes in detail the statistical methods used in the analysis of the reported data as stated in 11 above
		(*b*) Describe any methods used to examine subgroups and interactions	N/A	
		(*c*) Explain how missing data were addressed	N/A	
		(*d*) *Cohort study*—If applicable, explain how loss to follow-up was addressed<br>*Case-control study*—If applicable, explain how matching of cases and controls was addressed<br>*Cross-sectional study*—If applicable, describe analytical methods taking account of sampling strategy	N/A	
		(*e*) Describe any sensitivity analyses	N/A	
Results				
Participants	13*	(a) Report numbers of individuals at each stage of study—eg numbers potentially eligible, examined for eligibility, confirmed eligible, included in the study, completing follow-up, and analysed	7	The number of individuals at each stage of the study is provided in the paper -

				summarized in a study flow diagram (Fig. 1)
		(b) Give reasons for non-participation at each stage	N/A	
		(c) Consider use of a flow diagram	11	A flow diagram was used (Fig. 1)
Descriptive data	14*	(a) Give characteristics of study participants (eg demographic, clinical, social) and information on exposures and potential confounders	6-8	The report provides a summary of the characteristics of study participants.
		(b) Indicate number of participants with missing data for each variable of interest	4	Records of participants with significant missing data were excluded from the study.
		(c) *Cohort study*—Summarise follow-up time (eg, average and total amount)		
Outcome data	15*	*Cohort study*—Report numbers of outcome events or summary measures over time		
		Case-control study—Report numbers in each exposure category, or summary measures of exposure		
		Cross-sectional study—Report numbers of outcome events or summary measures	8 & 11	The report includes outcome events. Nineteen of the 74 children and adolescents with osteosarcoma had undergone manipulative therapy, giving a prevalence of 25.7%. The median survival for patients who underwent manipulative therapy was 1.0 year (95% CI 0.8-1.3), and it was 1.8 years (95% CI 1.4-2.2) for those who did not undergo manipulative therapy.
Main results	16	(*a*) Give unadjusted estimates and, if applicable, confounder-adjusted estimates and their precision (eg, 95% confidence interval). Make clear which confounders were adjusted for and why they were included	7 & 10	The report gives unadjusted estimates under the results section

	(*b*) Report category boundaries when continuous variables were categorized	NA
	(*c*) If relevant, consider translating estimates of relative risk into absolute risk for a meaningful time period	N/A

Continued on next page

Other analyses	17	Report other analyses done—eg analyses of subgroups and interactions, and sensitivity analyses	N/A	
**Discussion**				
Key results	18	Summarise key results with reference to study objectives	11-12	Pre-diagnosis manipulative therapy in children with osteosarcoma is an unexplored problem, especially in resource-limited settings, where the healthcare system is challenging. Our study demonstrated that pre-diagnostic manipulative therapy is prevalent among our child and adolescent populations with osteosarcoma and also indicated the characteristics associated with this practice, although the true impact on survival is unclear.
Limitations	19	Discuss limitations of the study, taking into account sources of potential bias or imprecision. Discuss both direction and magnitude of any potential bias	15	The limitation of this study is the possibility of reporting bias of any prior manipulation therapy, especially given that some of these were undocumented, some of which, like local cuttings and use of herbs, are believed to carry negative connotations in the conventional healthcare system. Likewise, the study was conducted in only one oncology unit in Uganda, and though the findings may not necessarily be generalizable to the whole region or other contexts, it underscores the challenges that are

				prevalent in similar-resource settings.
Interpretation	20	Give a cautious overall interpretation of results considering objectives, limitations, multiplicity of analyses, results from similar studies, and other relevant evidence	11-15	The report gives interpretation of the results in line with the objectives and limitations of the study.
Generalisability	21	Discuss the generalisability (external validity) of the study results	15	The study was conducted in only one oncology unit in Uganda, and though the findings may not necessarily be generalizable to the whole region or other contexts, it underscores the challenges that are prevalent in similar-resource settings.
**Other information**				
Funding	22	Give the source of funding and the role of the funders for the present study and, if applicable, for the original study on which the present article is based	16	This study did not receive any funding

*Give information separately for cases and controls in case-control studies and, if applicable, for exposed and unexposed groups in cohort and cross-sectional studies.

Note: An Explanation and Elaboration article discusses each checklist item and gives methodological background and published examples of transparent reporting. The STROBE checklist is best used in conjunction with this article (freely available on the Web sites of PLoS Medicine at http://www.plosmedicine.org/, Annals of Internal Medicine at http://www.annals.org/, and Epidemiology at http://www.epidem.com/). Information on the STROBE Initiative is available at www.strobe-statement.org.
